# Evaluating the Consistency of the 1982–1999 NDVI Trends in the Iberian Peninsula across Four Time-series Derived from the AVHRR Sensor: LTDR, GIMMS, FASIR, and PAL-II

**DOI:** 10.3390/s100201291

**Published:** 2010-02-08

**Authors:** Domingo Alcaraz-Segura, Elisa Liras, Siham Tabik, José Paruelo, Javier Cabello

**Affiliations:** 1 Departamento de Biología Vegetal y Ecología, Centro Andaluz para la Evaluación y Seguimiento del Cambio Global–CAESCG, Universidad de Almería, Ctra. Sacramento s/n. Almería, 04120, Spain; E-Mails: eliras@ual.es (E.L.); jcabello@ual.es (J.C.); 2 Laboratorio de Análisis Regional y Teledetección, Facultad de Agronomía and IFEVA, Universidad de Buenos Aires and CONICET. Avda. San Martín, 4453. Buenos Aires, 1417, Argentina; E-Mail: paruelo@ifeva.edu.ar; 3 Departamento de Arquitectura de Computadores, Universidad de Málaga, Campus de Teatinos, Apdo. 4114, 29080, Spain; E-Mail: stabik@uma.es

**Keywords:** seasonal Mann-Kendall trend test, temporal trends analysis, spatial statistics, partial Mantel test, carbon gains, Spain, Portugal, Iberian Peninsula

## Abstract

Successive efforts have processed the Advanced Very High Resolution Radiometer (AVHRR) sensor archive to produce Normalized Difference Vegetation Index (NDVI) datasets (*i.e.*, PAL, FASIR, GIMMS, and LTDR) under different corrections and processing schemes. Since NDVI datasets are used to evaluate carbon gains, differences among them may affect nations’ carbon budgets in meeting international targets (such as the Kyoto Protocol). This study addresses the consistency across AVHRR NDVI datasets in the Iberian Peninsula (Spain and Portugal) by evaluating whether their 1982–1999 NDVI trends show similar spatial patterns. Significant trends were calculated with the seasonal Mann-Kendall trend test and their spatial consistency with partial Mantel tests. Over 23% of the Peninsula (N, E, and central mountain ranges) showed positive and significant NDVI trends across the four datasets and an additional 18% across three datasets. In 20% of Iberia (SW quadrant), the four datasets exhibited an absence of significant trends and an additional 22% across three datasets. Significant NDVI decreases were scarce (croplands in the Guadalquivir and Segura basins, La Mancha plains, and Valencia). Spatial consistency of significant trends across at least three datasets was observed in 83% of the Peninsula, but it decreased to 47% when comparing across the four datasets. FASIR, PAL, and LTDR were the most spatially similar datasets, while GIMMS was the most different. The different performance of each AVHRR dataset to detect significant NDVI trends (e.g., LTDR detected greater significant trends (both positive and negative) and in 32% more pixels than GIMMS) has great implications to evaluate carbon budgets. The lack of spatial consistency across NDVI datasets derived from the same AVHRR sensor archive, makes it advisable to evaluate carbon gains trends using several satellite datasets and, whether possible, independent/additional data sources to contrast.

## 1. Introduction

Since the early eighties, the Advanced Very High Resolution Radiometer (AVHRR) sensors onboard the National Oceanic and Atmospheric Administration (NOAA) satellite series have been capturing daily images of the world, providing spectral information to monitor atmospheric, oceanic, vegetation, and land properties of the Earth. To date, three versions of the AVHRR sensor have operated: AVHRR/1 (with four channels, operating between 1979 and 1994 onboard the NOAA-6, -8, -10 satellites), AVHRR/2 (with five channels, operating between 1981 and 1999 onboard the NOAA-7, -9, -11, -12, -13, -14 satellites), and AVHRR/3 (with six channels, operating since 1999 to present onboard the NOAA-15, -16, -17, -18 satellites) (http://goespoes.gsfc.nasa.gov/poes/project/index.html, January 2010). A long-term (1981-present) time-series of global AVHRR daily images has been stored at degraded resolution in the Global Area Coverage (GAC) archive. The GAC images are a resample of the full 1.1 km resolution AVHRR images by averaging four out of every five samples along the scan line and processing only every third scan line. The final resolution is 1.1 × 4.4 km at the subpoint, although it is generally treated as 4 km resolution. Repeated efforts have processed the GAC archive attempting to produce datasets of consistent time-series of surface reflectance and spectral indices with enough quality to study the long-term dynamics and trends of different properties of the Earth. Despite the images were captured by similar AVHRR sensors, many issues have to be considered to avoid artifacts that may lead to missing or detecting trends in the time series that are or are not related to actual changes in important spectral properties of the Earth (e.g., [1,2]).

One of the important spectral indices that shows dissimilar long-term trends between different AVHRR-derived datasets is the Normalized Difference Vegetation Index (NDVI) (e.g., [3,4]). The NDVI is calculated from the reflectance in the AVHRR red (channel 1, 580–680 nm) and near infrared (channel 2, 725–1,100 nm) bands as follows [5,6]: NDVI = (NIR − R)/(NIR + R). This spectral index is strongly related to the fraction of the incoming photosynthetically active radiation intercepted by green vegetation [7] and it is widely and satisfactorily used for monitoring changes in ecosystem structure and function [8], detecting long-term trends in vegetation growth and phenology [9,10], providing inputs for primary production [11] and global circulation [12] models, and providing a reference to model the carbon balance worldwide [13–15].

Since the AVHRR sensor series were not originally designed for vegetation monitoring (but rather meteorological studies) and suffer from lack of onboard calibration and navigation/georeferencing problems, they have several shortcomings for this purpose [16–19]. To achieve a consistent NDVI time-series, the different processing efforts of the GAC archive had to deal with a wide range of factors affecting the NDVI. Van Leeuwen *et al*. [1] showed how multi-sensor NDVI time-series would significantly benefit if atmospheric corrections were adequately addressed. For instance, the AVHRR near-infrared band (channel 2) overlaps a wavelength interval in which there is considerable radiation absorption by water vapor in the atmosphere, which significantly decreases observed NDVI [20,21]. Other atmospheric corrections must also include ozone absorption, Rayleigh scattering, tropospheric aerosol optical thickness, and presence of aerosols in the stratosphere after major volcanic eruptions (e.g., El Chichón and Pinatubo). In addition to atmospheric corrections, the NDVI signal must be corrected for the variation in the solar zenith and viewing angles due to the orbital drift through the lifetime of the satellites [22]. Finally, AVHRR reflectance and NDVI data must also be corrected for sensor degradation and cross-calibration due to inter-sensor differences in spectral response functions of different sensor red and near-infrared bands [1]. In the case of the AVHRR GAC archive, an additional source of uncertainty may impact the quality of the data: it consists of the data reduction methodology used for transforming the 1.1 km resolution AVHRR data into the coarse resolution of the GAC archive [23,24].

Depending on the different corrections applied and processing streams and algorithms used, successive efforts have produced different coarse resolution AVHRR NDVI datasets from the original GAC data. The most common and broadly used ones are, from the earliest to the foremost, Pathfinder AVHRR Land (PAL I and II) [24,25], Fourier-Adjustment, Solar zenith angle corrected, Interpolated Reconstructed (FASIR) [26], Global Monitoring and Mapping Studies (GIMMS) [27], and Land Long-Term Data Record (LTDR) [28] datasets. Several studies have evaluated the consistency of the NDVI trends across the PAL, FASIR, and GIMMS AVHRR datasets in different regions of the world (e.g., [29,30]), and have also compared them to those derived from SPOT VEGETATION and MODIS Terra sensors (e.g., [31]). In some of these regions, the NDVI trends have been consistent across datasets and sensors, for instance, in the humid Sahel (but not in the driest; [31]), or the Chilean arid zones [4]. Contrary, in other regions, the use of different datasets has led to conflicting findings, potentially due to differences in the processing and corrections applied to the GAC data [e.g., 3,4,32,33]. Despite the LTDR dataset is the most recently produced one and incorporates much of the learning from the previous efforts, it does not still exist any published study that includes this dataset to evaluate the consistency of the NDVI trends.

This is also the case of the Iberian Peninsula (Spain and Portugal), where previous studies have calculated the NDVI trends based on AVHRR datasets at the regional [34–37] and local [38–40] scales, but none have evaluated their consistency across different datasets. Following up the suggestions of recent works [3,4,31], in this article we evaluate the spatial consistency of four AVHRR NDVI datasets to detect NDVI trends in the Iberian Peninsula, with a special focus on the recently released LTDR dataset. We also evaluated the error budget of the NDVI trends from the different slopes obtained across datasets. As far as we know, this is the first evaluation of the performance of the new LTDR dataset to detect NDVI trends.

## 2. Data and Methods

### 2.1. Satellite Datasets

We focused on the Iberian Peninsula to compare the 1982–1999 NDVI trends across four datasets derived from the GAC archive of the AVHRR sensor (NOAA-7, -9, -11, and -14 satellites; for a comparison of the datasets see Table 1 in this paper, and Table 1 in Baldi *et al*. [4]). We used the portion of the images located between 35° N and 45° N latitude, and 3.5° E and 10.2° W longitude. The period considered includes both extremely dry and wet periods for the Peninsula [41,42].

The first dataset was the Pathfinder AVHRR Land-II (PAL-II) dataset. It consists in 10-day NDVI composites at 64 km^2^ spatial resolution. Images were radiometrically and spatially corrected (for details see [24,43]). The atmospheric correction scheme follows the algorithm of Gordon *et al*. [43], including Rayleigh scattering and ozone. PAL-II did not correct for aerosols, water vapor, or satellite drift. The second dataset was the “Fourier-Adjustment, Solar zenith angle corrected, Interpolated Reconstructed” (FASIR, version 4.13) [26] dataset. Since it made use of the PAL-II dataset, it also has a spatial resolution of 64 km^2^, and contains 10-day composite images. In addition to PAL-II corrections, it includes Fourier adjustment of outliers and a bidirectional reflectance distribution function that seeks a common viewing and illumination geometry. The third dataset was obtained from the Global Inventory, Modeling and Mapping Studies (GIMMS) team and includes the new and updated release of the per-continent global data (1981–2006) made available in 2007 [44]. Currently, the GIMMS dataset is the most commonly used dataset to model and evaluate vegetation patterns and trends around the world. It has a spatial resolution of 64 km^2^, and contains two composite images per month. It has been corrected for sensor degradation, inter-sensor differences, solar zenith angle and viewing angle effects due to satellite drift (using an empirical mode decomposition function [22]), cloud cover, volcanic aerosols, and other effects not related to vegetation change (it is not corrected for water vapor, ozone, and scattering) (for details see [27]). GIMMS is currently thought to be consistent with NDVI derived from VEGETATION and Moderate-Resolution Imaging Spectroradiometer (MODIS) sensors [27].

The former three NDVI datasets have also been recently compared by Baldi *et al*. [4] for South America (see Table 1 in Baldi *et al*. [4] for a detailed comparison). Our study, in addition, makes use of the newest AVHRR dataset created by the Land Long Term Data Record (LTDR) team [28] (in Table 1, we provide an extension of Baldi *et al*. [4] table for the LTDR version 2 dataset). LTDR is a NASA-funded REASoN project that aims to produce a consistent long term data set from AVHRR, MODIS, and Visible/Infrared Imager/Radiometer Suite (VIIRS) sensors. The LTDR project is reprocessing GAC data from 1981-present by applying the preprocessing improvements identified in the Pathfinder AVHRR Land II (PAL-II) project, and the atmospheric and BRDF corrections used in MODIS preprocessing steps (http://www.ltdr.nascom.nasa.gov, September 2009) [28]. The LTDR dataset consists in daily global images at a spatial resolution of 0.05 × 0.05 degrees (∼5 km^2^). As in the former datasets, we calculated 15-day maximum value composites [20] of the LTDR daily images to minimize the noise due to cloud cover, cloud shadow, and aerosols contamination (though it may not be completely removed [2]). Despite the geolocation accuracy is supposed to be of about one pixel, in our evaluation of the LTDR version 2 dataset, we have found a long-term systematic geolocation displacement of 2 to 3 pixels from the NW to the SE of the images along the 1982–1999 period. This caused an “artificial” NDVI negative trend in the NW border of the continents, and a positive NDVI trend in the SW borders (Alcaraz-Segura, unpublished).

### 2.2. Temporal Trend Analysis

Since NDVI time-series often do not meet parametric assumptions such as normality and homoscedasticity, we evaluated the existence of significant 1982–1999 NDVI trends by using the seasonal Mann-Kendall trend test (as suggested by de Beurs and Henebry [9]). This is a rank-based non-parametric test robust against seasonality, non-normality, heterocedasticity, missing values, and intra-annual autocorrelation [51–53]. The use of this test avoids the loss of seasonal information when checking for trends, so we kept the full temporal resolution of the NDVI seasonal dynamics in all datasets (instead of using just the annual mean or maximum).

The seasonal Mann-Kendall test first evaluates whether each periodic sub-annual interval (*i.e.*, months, composite periods, or seasons) exhibits significant monotonic trends based on Kendall’s S score and its variance. Then it computes a Z score and performs a heterogeneity test to see if this trend is consistent across all sub-annual intervals. To minimize the influence of errors, outliers, missing data, and tied observations on the slope estimation [54], we used a non-parametric linear slope estimator suggested by Sen [55]. First, in each sub-annual period, Sen’s Method calculates the median of all possible two-point slopes between pairs of years [51] but discarding tied observations [56]. Then, it calculates the median of all the sub-annual period slopes [54].

The trend test was run using the MATLAB code “Seasonal Kendall Test with Slope for Serial Dependent Data” provided by Jeff Burkey through the MATLAB Central file exchange (http://www.mathworks.com, accessed May 2009). At present, this test is corrected for intra-annual autocorrelation but not for inter-annual autocorrelation [57]. For each pixel and dataset, the overall slope obtained with the Sen Method, and the p-value calculated with the seasonal Mann-Kendall trend test, were stored. Significant slopes were assumed for p-values < 0.05.

### 2.3. Spatial Consistency Analysis

The slope and p-value images of the four datasets were transformed to a common UTM 30N projection, European 1950 Datum, and 8 km pixel size. Then, we removed from the analysis all pixels that were considered as sea in the PAL, FASIR, or GIMMS datasets, or as water in the quality assessment flag of the LTDR dataset. Three types of analyses were carried out to evaluate the spatial consistency of the NDVI trends across the four datasets. First, we created a consensus map that displays for every pixel the degree of consistency across the four datasets (Table 2). Second, we compared across datasets the percentage of pixels showing significant trends, the polarity of the trends, and the magnitude of their slopes. We also evaluated the relative error budget of the NDVI trends by calculating the coefficient of variation of the slopes across the four datasets.

Finally, partial Mantel tests (an evaluation of spatial similarity) [58–60] were used to examine the correlation between pairs of AVHRR datasets while controlling the effect of spatial autocorrelation to remove spurious correlations [61] (*i.e.*, accounting for the influence that the spatial autocorrelation of trends among proximate pixels has on the calculation of the correlation between AVHRR datasets). The partial Mantel statistic calculates the partial Pearson correlation between the two dissimilarity matrices (A and B, one for each AVHRR dataset trends) conditioned by a third dissimilarity matrix (C, the geographical distance between pixel locations). Each dissimilarity matrix corresponds to a symmetric n x n matrix where rows and columns corresponded to the same sampled pixels (n) and where the value of each i,j cell was the Euclidian distance (difference) between the i row pixel and the j column pixel (expressing difference in the slope of the NDVI trend for A and B, and geographical distance for C). If matrix C (“space”) is not related to matrices A and B (“datasets”), we simply would get the Pearson correlation coefficient between A and B. The significance of the relationship was evaluated by permuting (1,000 times) rows and columns in the first dissimilarity matrix (A), but keeping constant the other two [62]. The test was run using the “Community Ecology vegan 1.15–4” R-package [63]. Due to computational limitations, we bootstrapped the analysis 10,000 times, using 100 pixels showing significant trends as sample size for each comparison. In addition, following the same bootstrapping procedure, we also calculated Pearson’s correlation coefficients to compare the linear relationship among the datasets when spatial autocorrelation was not expressly accounted for in the analysis. To look for significant differences in linear correlation (Pearson’s r) and spatial similarity (partial Mantel’s r) among the four datasets, we ran 20 ANOVA tests using random subsamples of 50 significant (p-value < 0.001) values of r from the 10,000 bootstrapped analyses. Comparisons between classes were based on the Sheffe’s S procedure, which provides a confidence level (alpha of 0.05) for comparisons of means among all datasets, and it is conservative for comparisons of simple differences of pairs. All analyses were repeated considering all NDVI trends (significant and non-significant) altogether, whose results are shown in the Appendices.

## 3. Results

**Figure 1.**
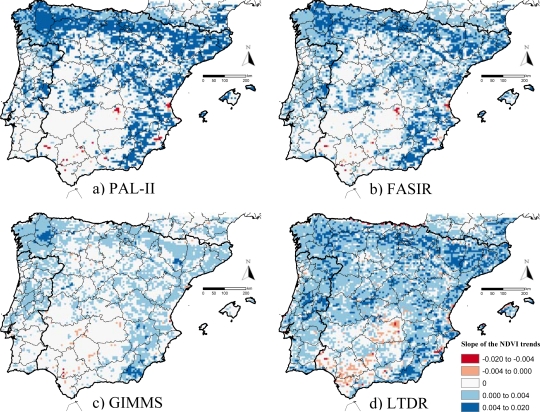
Difference in magnitude and spatial patterns of the significant 1982–1999 NDVI trends across four AVHRR datasets: (a) PAL-II, (b) FASIR, (c) GIMMS, and (d) LTDR for the Iberian Peninsula. Significant trends were considered for p-values < 0.05 by means of the seasonal Mann-Kendall trend test. Slopes express change of NDVI per year.

The AVHRR datasets showed that most of Iberia experienced either positive or no trends in NDVI during the 1982–1999 period. The areas with negative trends are small and isolated, despite the dataset considered (Figure 1, Table 4). Datasets, though, differed in the magnitude of the NDVI trends (Figure 1, Table 3). The mean NDVI trend of the whole Iberian Peninsula was similar and positive for PAL-II, FASIR, and LTDR datasets (Table 3), though it was half in magnitude for the GIMMS dataset. When the means for positive and negative significant trends were calculated separately, the PAL-II dataset showed the steepest trends (Table 3), while the GIMMS datasets showed the weakest ones (half in magnitude than PAL-II).

**Figure 2.**
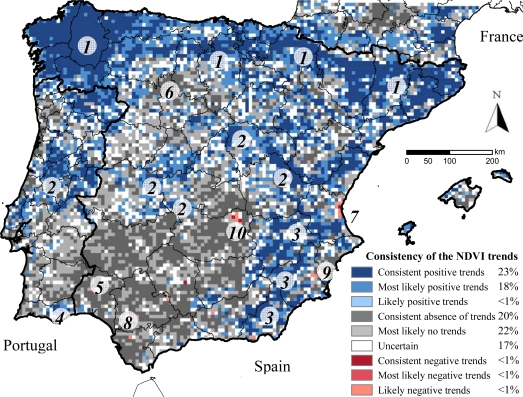
Consensus map showing the spatial consistency of the significant NDVI trends across the four datasets (PAL-II, FASIR, GIMMS, and LTDR) for the Iberian Peninsula. See Table 2 for legend explanation. Significant trends were considered for p-values < 0.05 by means of the seasonal Mann-Kendall trend test. Percentages in the legend indicated the extension of each class in the Peninsula. Locations referred in the text: (1) northern, (2) central, (3) southeastern, (4) Algarve, and (5) Aracena mountain ranges; croplands of: (6) the Duero basin plains, (7) Valencia, (8) Guadalquivir basin, (9) Segura basin, and (10) La Mancha plains.

The consensus map (Figure 2) showed that in 20% of the Peninsula the four datasets exhibited absence of significant trends (pixels distributed across the southwestern quarter of the Peninsula and the agricultural high plains of the Duero basin) (Table 4).

In an additional 22% of Iberia, three databases did not detect significant trends (most likely no trends in Figure 2). A 23% of the Peninsula showed positive and significant NDVI trends across the four datasets (areas along the northern, central, and southeastern mountain ranges) and an additional 18% across three databases (most likely positive trends in Figure 2). Consistent significant negative NDVI trends across the four datasets occurred in less than 1% of the Peninsula (isolated pixels in Aracena mountains and in agricultural areas in the Guadalquivir and Segura basins, La Mancha plains, and Valencia). The LTDR dataset showed the greatest percentage of pixels with significant NDVI trends (Table 4), while the GIMMS dataset showed the lowest (LTDR detected significant trends in 32% more pixels than GIMMS) (Table 4). In Spain, the percentage of pixels exhibiting consistent significant NDVI trends across all datasets was 7.8% greater than in Portugal, though it varied depending on the dataset (e.g., for PAL, Spain showed 15% more pixels with significant trends than Portugal, while for LTDR, Portugal showed 6.7% more trending pixels than Spain) (Table 4). The relative error budget of the NDVI trends, evaluated by means of the coefficient of variation of the slopes across the four datasets, showed a similar spatial pattern to the consensus map. Those areas showing consistent positive or negative significant trends across the four datasets differed on average by less than 50% in the magnitude of their slopes, while in areas showing non-significant trends, the magnitude and sense of the slopes largely varied across datasets by more than 100%.

**Figure 3.**
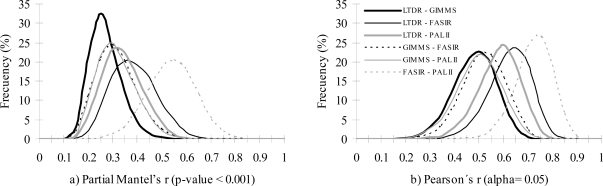
Comparison of the (a) spatial similarity (partial Mantel’s r) and (b) correlation (Pearson’s r) of the significant NDVI trends (p-value < 0.05) between pairs of the four AVHRR datasets in the Iberian Peninsula. The frequency histograms of Pearson’s and partial Mantel’s r resulted from 10000 bootstrapped tests using random subsamples of 100 pixels. Significant values of r were considered for p-values < 0.001.

From the four AVHRR datasets, spatial similarity and correlation of significant NDVI trends was the greatest between FASIR and PAL datasets, and the lowest between GIMMS and LTDR (Table 5, Figure 3). The rest of the comparisons of the spatial distribution of significant NDVI trends showed comparable partial Mantel’s r and Pearson’s r (Figure 3), and percentage of consensus in the contingency table (Table 5). The GIMMS dataset showed the lowest correlation with the other three datasets, while the LTDR datasets showed a moderate correlation with PAL-II and FASIR (but not with GIMMS dataset).

## 4. Discussion and Conclusions

Coarse-resolution satellite datasets of the NDVI derived from the AVHRR GAC archive are one of the most valuable sources to evaluate temporal trends of carbon gains at the global, regional, and national scales. In carbon budget assessments, countries often make use of these satellite datasets to estimate both vegetation uptake and land-use change related release [14]. In our study for the Iberian Peninsula, the AVHRR datasets clearly showed that the area showing significant positive NDVI trends is important (23% and an additional 18% of Iberia showed consistency across four and three datasets respectively) and much larger than the proportion with decreasing trends (only 0.1% of Iberia). The area without significant trends was also important (consistently in 20% and an additional 22% of Iberia across four and three datasets respectively). However, although clear consistent patterns may emerge at the country level or regional scale, local analyses must consider that the area showing significant trends can vary depending on the analyzed dataset. For instance, in the whole Iberian Peninsula, it varied from 37 to 67% of the area for positive trends, and from 0.6 to 3.6% for negative trends, and these differences were much larger for Portugal than for Spain (Table 4). Our study quantified a large portion of the territory (57% of pixels for the Peninsula, 66% for Portugal, and 55% for Spain) where the use of different NDVI datasets may lead to inconsistent NDVI significant trends (though it decreased to just 30%, 37%, and 38% respectively when only the sign of the slope but not the significance was considered (Appendix 3)). For agreements across just two datasets (contingency tables of Table 5 and Appendix 5), the spatial inconsistency was much lower (even just 20% in the comparison between PAL and FASIR; Table 5). In addition to the differences in the magnitude of the NDVI trends between GIMMS and LTDR (Table 3), they showed the lowest percentage of agreement in the contingency table (Table 5). However, their spatial consistency largely increased when non-significant slopes were also compared (Appendix 5), due to the lower sensitivity of GIMMS to detect both positive and negative NDVI trends.

Regarding the spatial distribution of the NDVI trends in the Iberian Peninsula, increases in vegetation greenness (consistent and most likely positive trends) were largely observed along mountain ranges in the north, center, and southeast of both Spain and Portugal, mainly occupied by natural forests and tree plantations. This increase in the photosynthetic activity agrees with the general trend observed in Europe due to the increase in the forested area, the juvenile age structure, CO_2_-fertilization, elevated atmospheric nitrogen deposition, and climate change [14]. NDVI increases were also aligned along the Ebro river margins, where irrigation expansion over drylands has increased productivity [34,36]. Decreases in vegetation greenness were scarce and localized mainly on agricultural lands along the southern and eastern river valleys (Figures 1 and 2, and Appendixes 1 and 3) and were largely related to land-use changes on croplands: in Valencia, NDVI decrease was related to urban expansion and *Citrus* crop abandonment [36,37]; in La Mancha, it was related to the abandonment of vineyards and unsustainable irrigation due to the drop of groundwater tables [36,64]; in the Segura river valley, it may be due to both urban expansion and abandonment of unsustainable irrigation [64,65]; in the Guadalquivir river valley, NDVI decreases seem to be caused by a decrease in irrigation and rainfall both originated by lower precipitations determined by a trend towards positive phases of the North Atlantic Oscillation (NAO) [34,37]. In the woodlands of Sierra de Aracena, NDVI decreases also seem to be caused by lower precipitations related to the NAO dynamics [34,37]. The regional control of the NAO dynamics over the NDVI trends of the southwestern quadrant is also suggested by the high local spatial autocorrelation in this region (Appendix 7), which should be further investigated. The PAL, FASIR, and LTDR (but not GIMMS) datasets also displayed high spatial autocorrelation in the north and northeastern regions and along the river Ebro valley. Only the GIMMS dataset showed very strong autocorrelation in NW Spain (Appendix 7).

Many factors may be responsible for the retrieval of different significant NDVI trends across datasets, such as differences among their corrections schemes, projection systems, temporal resolution, or geolocation errors. For instance, the PAL dataset is known to be strongly affected by both satellite drift and volcanic aerosols, while GIMMS does not explicitly address atmospheric corrections [2], and LTDR still lacks complete atmospheric correction [2]. In the case of temporal resolution, datasets with longer composite periods (e.g., GIMMS and LTDR) are less affected by cloud noise [20] but, since they also have fewer composites per year, they may offer less power to retrieve significant trends. Hence, in our analysis, it would be expected to have less power in the retrieval of significant trends using datasets with fewer composites such as GIMMS and LTDR (24 composites per year), than with more composites such as PAL and FASIR (36 composites per year). However, this only happened with the GIMMS dataset, the one showing the lowest percentage of significant trends, while the LTDR dataset cumulated the greatest percentage of significant trends (Table 4). Additionally, the differences in projections and geolocation error, seem to be partially responsible of the very low spatial correspondence of significant negative NDVI trends since they were mainly local (occupying a few pixels) and along river valleys (Figure 1, Appendix 1). From our findings, in addition to quantifying the area affected by consistent trends in vegetation greenness, carbon budget evaluations should also assess the differences in magnitude of the NDVI trends, which can largely vary across datasets. In the case of Spain and Portugal, the maximum difference across datasets was more than double both for the global mean, and for positive and negative trends separately.

C gains estimates had historically relied on forest inventories or land use–land cover changes [15,66,67]. Remotely sensed data has been incorporated as a tool to derive C gains for non-forested areas and for areas without previous inventories [68–70]. From our and previous studies [3,4], it is recommended that evaluations of the carbon balance based on regional NDVI trends derived from coarse-resolution AVHRR sensor datasets are compared across several datasets to minimize the broad effects that potential local or regional biases in one of them may cause into national carbon budgets. Currently, the PAL and FASIR datasets have been mostly substituted by the GIMMS dataset as reference to model the carbon balance worldwide [13,15]. Since the LTDR dataset has been produced as the first component of a cross-sensor long-term NDVI record (to be continued by the MODIS and VIIRS sensors), it is expected that LTDR will also replace all previous AVHRR datasets in this type of studies. However, in our analysis, GIMMS and LTDR, the two “most improved” and newest AVHRR global datasets, showed the lowest consistency between each other. This strongly suggests that the LTDR NDVI trends should also be compared across several AVHRR datasets and, ideally, with independent sensors (such as VEGETATION SPOT or MODIS) to seek for consistencies that reduce as much uncertainty as possible. Future long-term NDVI datasets (e.g., coming versions of LTDR) should contain global estimates of their errors (as Nagol *et al*. also suggest [2]) and, whether possible, spatially and temporally explicit estimates of uncertainty. As an example of the spatial differences in error budgets, Appendix 8 expresses the relative uncertainty of the 1982–1999 NDVI trends throughout the Iberian Peninsula as the absolute value of the coefficient of variation of the NDVI slope across the four AVHRR datasets. Despite our error analysis being incomplete, it gives a sense of the relative level of uncertainty to consider when using NDVI trends to estimate carbon gains at the regional level.

A proper evaluation of satellite datasets should not only restrict to the physical and mathematical assumptions of image processing but it should also test them at the level of predictions, e.g., comparing trends derived from spectral data with independent observation of change [3,4]. Identified areas with extreme land cover changes that cover a substantial portion of a 64 km^2^ pixel (e.g., deforested areas in South America and expansion of center pivot agricultural systems over drylands) are ideal places to contrast remotely sensed trends with observed changes.

## Figures and Tables

**Table 1.** Description of the AVHRR LTDR NDVI dataset [2,28] used in this study (this table extends the comparison across PAL-II, FASIR, and GIMMS provided by Table 1 in Baldi *et al*. [4]).

	***LTDR version 2***
*Data Set Origins (and its spatial resolution)*	NOAA-AVHRR GAC L1B (1.1 × 4.4 km, known as 4 km)
*Instrument and change in times*	NOAA-7, -9, -11, -14 (to be expanded in next versions)
*Known temporal span*	1981–1999 (to be expanded in next versions)
*Temporal resolution*	The original dataset consists of daily images with no temporal compositing.
*Spatial resolution*	0.05 × 0.05 degrees, same as MODIS Climate Modeling Grid products
*Spatial compositing*	Forward, nearest neighbour mapping. Selection of the 4.4 km pixel with the maximum NDVI value for the 0.05° output bin. Only zenith angles less than 42° were used.
*Temporal compositing*	The original dataset consists of daily images with no temporal compositing.
*Radiometric corrections*	Ocean-clouds vicarious calibration using the Vermote/Kaufman parameters [45]. This technique uses ocean observations to track the degradation of channel 1 and observations of clouds to follow the evolution of the channel 1/channel 2 ratio. This vicarious calibration technique was evaluated [46] by using MODIS observations over a stable desert site, where the independently derived sets of AVHRR calibration coefficients were consistent to within less than 1%).
*Viewing and illumination corrections*	Correction of illumination and viewing angle effects with Bidirectional Reflectance Distribution Function (BRDF) techniques will be implemented in version 3.
*Cloud corrections*	Rigorous cloud (and cloud shadow) screening using Cloud Advanced Very High Resolution Radiometer (CLAVR-1) [23,44].
*Stratospheric aerosols correction*	Aerosol corrections will be implemented in version 3 [47].
*Molecular absorption and scattering corrections*	Rayleigh scattering and water vapour corrections based on Reanalysis ancillary data (surface pressure, water vapour, wind speed) from the NOAA Center for Environmental Prediction (NCEP) (surface pressure was refined with NOAA TBASE Digital Elevation Model) [48]. Ozone correction used concentration measurements from the Total Ozone Mapping Spectrometer (TOMS) [49]. Aerosol corrections will be implemented in version 3 [47].
*Manual checking*	On navigation accuracy, data drop outs, bad scan lines, and other strange values. Inverse navigation to relate an Earth location to each sensor instantaneous field of view.
*Noise attenuation*	No specific noise attenuation applied.
*Scaling procedures*	No specific scaling procedures applied.
*Quality Assessment (QA)*	MODIS-like [50] 16 QA bits must be used prior to using a given pixel in any scientific analysis.
*Errors*	- *Geolocation*: ∼1 pixel accuracy. Orbital model run with corrected on-board clock and ephemeris data and inverse navigation to geolocate each sensor’s instantaneous field of view [28].
	- *NDVI*: Accuracy: 0.0064 to 0.024; Precision: 0.02 to 0.037 (for clear and average atmospheric conditions) [2]. RMS error about the one-to-one line between daily NDVI images and the NDVI calculated at 48 AERONET sites in 1999 is two times lower for LTDR than for PAL [28].

**Table 2.** Legend of the consensus map of Figure 2 displaying the degree of consistency of the NDVI trends across the four datasets. The possible combinations were classified into the following nine categories.

	**Full consistency**	**Most likely**	**Ambiguous**
**Positive significant trends**	**1**: all datasets show significant positive trends	**2**: three datasets agree, one shows absence of significant trends	**3**: two datasets agree, two show absence of significant trends
**Absence of significant trends**	**4**: all datasets show absence of significant trends	**5**: three datasets agree, one shows significant trends	**6**: remaining combinations (uncertain trends)
**Negative significant trends**	**7**: all datasets show significant negative trends	**8**: three datasets agree, one shows absence of significant trends	**9**: two datasets agree, two show absence of significant trends

**Table 3.** Differences in the magnitude of the 1982–1999 NDVI significant trends across four AVHRR datasets for the Iberian Peninsula. Significant trends were considered for p-values < 0.05 by means of the seasonal Mann-Kendall trend test. Slopes express change of NDVI per year.

	**PAL-II**	**FASIR**	**GIMMS**	**LTDR**
*Greatest significant positive slope*	0.0144	0.0116	0.0066	0.0136
*Greatest significant negative slope*	−0.0069	−0.0066	−0.0057	−0.0178
*Global mean slope (including zeros)*	0.0019	0.0018	0.0009	0.0020
*Mean of significant positive trends*	0.0045	0.0037	0.0024	0.0031
*Mean of significant negative trends*	−0.0044	−0.0037	−0.0023	−0.0027

**Table 4.** Percentage of pixels of Spain, Portugal, and the Iberian Peninsula exhibiting significant NDVI trends (p-value < 0.05) in each AVHRR dataset (PAL-II, FASIR, GIMMS, and LTDR), and percentage of pixels that showed consistent significant (positive, absence, or negative) NDVI trends across all four datasets. Significant NDVI trends were considered for p-values < 0.05 by means of the seasonal Mann-Kendall trend test.

	
	*% of pixels with:*	**PAL-II**	**FASIR**	**GIMMS**	**LTDR**	**Across all**
***SPAIN***	*Significant positive trends*	44.6	50.6	38.2	66.2	24.9
	*Non-significant NDVI trend*	54.8	48.5	60.9	29.8	20.4
	*Significant negative trends*	0.6	0.9	0.9	4.0	0.1

***PORTUGAL***	*Significant positive trends*	30.2	44.6	37.2	76.0	17.2
	*Non-significant NDVI trend*	69.8	55.3	62.6	23.0	17.0
	*Significant negative trends*	0.0	0.1	0.2	0.9	0.0

***IBERIAN PENINSULA***	*Significant positive trends*	42.4	48.8	37.2	67.0	22.6
	*Non-significant NDVI trend*	57.0	50.5	62.0	29.4	20.2
	*Significant negative trends*	0.6	0.7	0.8	3.6	0.1

**Table 5.** Contingency table showing the consensus across datasets in the number (lower left) and percentage (upper right) of pixels that exhibited significant NDVI trends (Negative or Positive) or non-significant trends (Absence) (p-value < 0.05). Darker gray tones highlight the highest consensus values.

%		**PAL**			**FASIR**			**GIMMS**			**LTDR**		

pixels		Negative	Absence	Positive	Negative	Absence	Positive	Negative	Absence	Positive	Negative	Absence	Positive
**PAL**	Negative				0.27	0.29	0.00	0.10	0.43	0.02	0.37	0.17	0.02
	Absence				0.43	43.89	12.70	0.60	45.31	11.11	3.00	24.86	29.17
	Positive				0.00	6.30	36.12	0.07	16.24	26.11	0.20	4.49	37.73

**FASIR**	Negative	27	44	0				0.09	0.60	0.01	0.50	0.19	0.01
	Absence	29	4440	637				0.54	41.40	8.53	2.81	24.03	23.64
	Positive	0	1285	3654				0.14	19.99	28.70	0.25	5.30	43.28

**GIMMS**	Negative	10	61	7	9	55	14				0.21	0.38	0.19
	Absence	44	4584	1643	61	4188	2022				3.21	25.07	33.71
	Positive	2	1124	2641	1	863	2903				0.14	4.07	33.03

**LTDR**	Negative	37	303	20	51	284	25	21	325	14			
	Absence	17	2515	454	19	2431	536	38	2536	412			
	Positive	2	2951	3817	1	2391	4378	19	3410	3341			

**Appendix 1.**
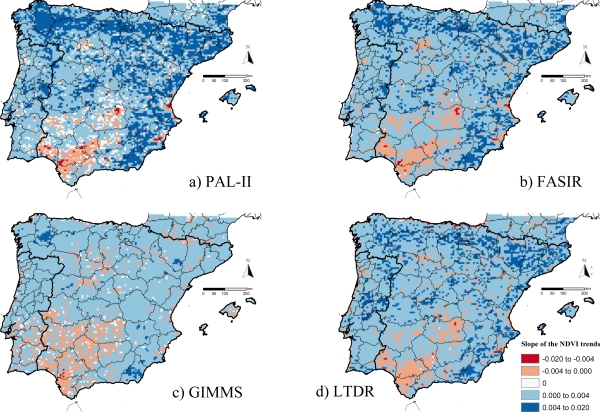
Difference in the magnitude and the spatial patterns of the 1982–1999 NDVI trends (both significant and non significant) across four AVHRR datasets: a) PAL-II, b) FASIR, c) GIMMS, and d) LTDR for the Iberian Peninsula (Spain and Portugal). Slopes express change of NDVI per year.

**Appendix 3.**
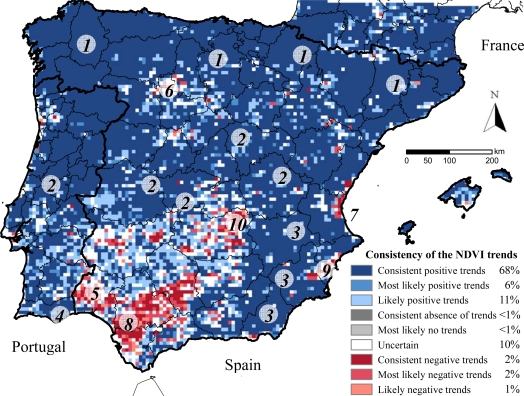
Consensus map showing the spatial consistency of the NDVI trends (both significant and non-significant) across the four datasets (PAL-II, FASIR, GIMMS, and LTDR) in the Iberian Peninsula. See Table 2 for legend explanation. Locations referred in the text: (1) northern, (2) central, (3) southeastern, (4) Algarve, and (5) Aracena mountain ranges; croplands of: (6) the Duero basin plains, (7) Valencia, (8) Guadalquivir basin, (9) Segura basin, and 10) La Mancha plains.

**Appendix 6.**
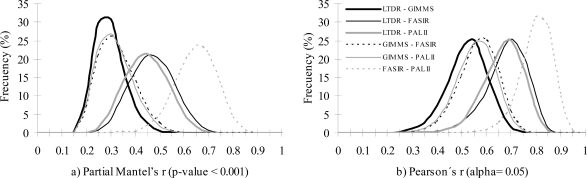
Comparison of the a) spatial similarity (partial Mantel’s r) and b) correlation (Pearson’s r) of the NDVI trends (both significant and non-significant) between pairs of the four AVHRR datasets in the Iberian Peninsula. The frequency histograms of Pearson’s and partial Mantel’s r resulted from 10000 bootstrapped tests using random subsamples of 100 pixels. Significant values of r were considered for p-values < 0.001.

**Appendix 7.**
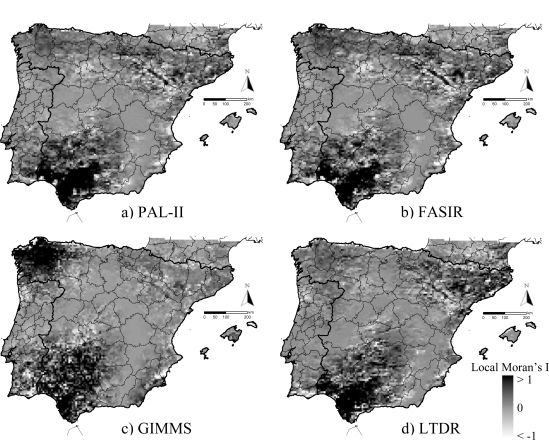
Geographical pattern of spatial autocorrelation (significant Local Moran’s I at p-value <0.05) of the 1982–1999 NDVI trends (both significant and non-significant) for the: (a) PAL-II, (b) FASIR, (c) GIMMS, and (d) LTDR datasets in the Iberian Peninsula.

**Appendix 8.**
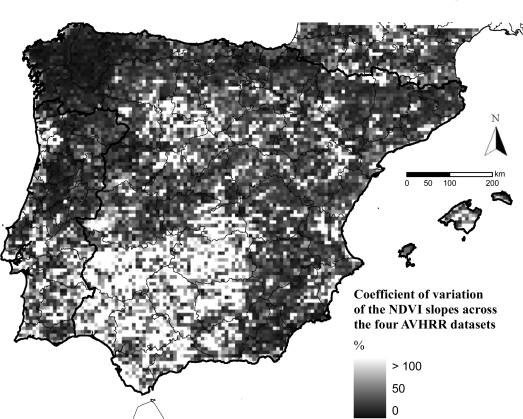
Map of the absolute value of the coefficient of variation of the NDVI slope across the four AVHRR datasets (PAL, FASIR, GIMMS, and LTDR) expressing the relative uncertainty of the 1982–1999 NDVI trends throughout the Iberian Peninsula (the lower the coefficient of variation, the lower the uncertainty of the NDVI slope).

## Appendices

### Appendix

**Appendix 2.** Differences in the magnitude of the 1982–1999 NDVI trends (both significant and non-significant) across four AVHRR datasets for the Iberian Peninsula (Spain and Portugal). Slopes express change of NDVI per year.

	**PAL-II**	**FASIR**	**GIMMS**	**LTDR**
*Greatest positive slope*	0.0144	0.0116	0.0066	0.0136
*Greatest negative slope*	−0.0069	−0.0066	−0.0057	−0.0178
*Global mean slope*	0.0027	0.0022	0.0012	0.0021
*Mean of positive trends*	0.0033	0.0027	0.0017	0.0026
*Mean of negative trends*	−0.0019	−0.0012	−0.0008	−0.0013

### Appendix

**Appendix 4.** % of pixels of Spain, Portugal, and the Iberian Peninsula exhibiting NDVI trends (both significant and non-significant) in each AVHRR dataset (PAL-II, FASIR, GIMMS, and LTDR), and % of pixels that showed consistent (positive, absence, or negative) NDVI trends across all four datasets.

	
	*% of pixels with:*	**PAL-II**	**FASIR**	**GIMMS**	**LTDR**	**Across all**
***SPAIN***	*Positive trends*	82.9	82.0	72.8	80.9	59.8
	*No NDVI trend*	9.3	9.3	17.7	11.2	0.8
	*Negative trends*	7.8	8.7	9.6	7.9	1.6

***PORTUGAL***	*Positive trends*	90.1	86.0	72.2	91.1	62.7
	*No NDVI trend*	7.8	11.8	20.3	5.7	0.5
	*Negative trends*	2.1	2.3	7.5	3.1	0.1

***IBERIAN PENINSULA***	*Positive trends*	84.6	86.4	81.7	88.2	68.1
	*No NDVI trend*	8.6	3.4	3.8	0.1	0.1
	*Negative trends*	6.8	10.2	14.5	11.7	2.1

### Appendix

**Appendix 5.** Contingency table showing the consensus across datasets in the number (lower left) and percentage (upper right) of pixels that exhibited NDVI trends (Negative or Positive) or no trends (Absence). Darker gray tones highlight the highest consensus values.

%		**PAL**			**FASIR**			**GIMMS**			**LTDR**		

pixels		Negative	Absence	Positive	Negative	Absence	Positive	Negative	Absence	Positive	Negative	Absence	Positive
**PAL**	Negative				3.07	2.33	0.00	3.95	1.29	0.16	4.58	0.78	0.05
	Absence				0.33	2.20	2.15	0.59	2.24	1.84	1.29	2.42	0.96
	Positive				0.00	1.71	88.21	0.30	4.02	85.60	2.16	3.28	84.48

**FASIR**	Negative	311		0				2.73	0.60	0.07	2.57	0.76	0.07
	Absence	236	223	173				1.68	1.66	2.91	3.76	1.69	0.80
	Positive	0	217	8923				0.43	5.30	84.62	1.70	4.03	84.62

**GIMMS**	Negative	400	60	30	276	170	44				3.40	0.90	0.54
	Absence	131	227	407	61	168	536				2.50	4.70	0.37
	Positive	16	186	8659	7	294	8560				2.13	0.89	84.58

**LTDR**	Negative	463	131	218	260	380	172	344	253	215			
	Absence	79	245	332	77	171	408	91	475	90			
	Positive	5	97	8546	7	81	8560	55	37	8556			

### Appendix

Appendix 7 shows an evaluation of the spatial structure of the NDVI slopes to show, for each dataset, those regions experiencing similar NDVI trends. We used the Local Moran’s I [71] to measure the local spatial autocorrelation of the NDVI slopes for each pixel considering all surrounding pixels within its area of influence (200 km). Moran’s I generally varies between −1 and 1, indicating negative or positive spatial autocorrelation respectively (though these limits can be exceeded [72]). Values near zero correspond to absence of autocorrelation [73]. The radius of influence was estimated as the distance where the spatial autocorrelation was no longer significant (p-value <0.05) by using correlograms, which measure the data autocorrelation as a function of the spatial distance [74].
